# Occupation in Insanity

**Published:** 1845-09

**Authors:** 


*Occupation in Insanity.*—At the recent anniversary of the Union Discipline Society in Boston, the following anecdote was related of a boy who was sent to the Brattleboro' Asylum, in a state of derangement. He told the physician he wanted work; he could'nt live without it. The doctor asked him what he could do? He could print—but they had no means of printing. "Well," he said, "the doctor could get work for him at the presses in the village." The doctor applied, but the printer said the boy would "knock the type all into *pi.*" The doctor told the boy the result of his application. "Well," said he, "I should do no such thing; but, doctor, you can buy a press; it will cost but little." The doctor made the experiment, bought press and paper, and enlisted the insane to contribute, edited the "Monthly Asylum Journal," and sent it abroad. The boy printed it, improved rapidly, and has entirely recovered,—and in the last Journal returned his thanks to the community for this means of restoration.—*N. Y. Exp.*

